# Accuracy of Alpha Amylase in Diagnosing Microaspiration in Intubated Critically-Ill Patients

**DOI:** 10.1371/journal.pone.0090851

**Published:** 2014-03-06

**Authors:** Florent Dewavrin, Farid Zerimech, Alexandre Boyer, Patrice Maboudou, Malika Balduyck, Alain Duhamel, Saad Nseir

**Affiliations:** 1 Intensive Care Unit, Valenciennes Hospital, avenue Desandrouin, Valenciennes, France; 2 Biochemistry and Molecular Biology Laboratory, Faculty of Pharmacy, Lille II University, Lille, France; 3 Medical Intensive Care Unit, CHU Bordeaux, Place Amélie Raba Léon, Bordeaux, France; 4 Epidemiology, Public Health and Quality of Care, Nord-de-France University, Lille, France; 5 Critical Care Center, R. Salengro Hospital, University Hospital of Lille, Lille, France; 6 Medical Assessment Laboratory, EA 2694, University of Lille Nord de France, Lille, France; University of Leicester, United Kingdom

## Abstract

**Objectives:**

Amylase concentration in respiratory secretions was reported to be a potentially useful marker for aspiration and pneumonia. The aim of this study was to determine accuracy of α-amylase in diagnosing microaspiration in critically ill patients.

**Methods:**

Retrospective analysis of prospectively collected data collected in a medical ICU. All patients requiring mechanical ventilation for at least 48 h, and included in a previous randomized controlled trial were eligible for this study, provided that at least one tracheal aspirate was available for α-amylase measurement. As part of the initial trial, pepsin was quantitatively measured in all tracheal aspirates during a 48-h period. All tracheal aspirates were frozen, allowing subsequent measurement of α-amylase for the purpose of the current study. Microaspiration was defined as the presence of at least one positive tracheal aspirate for pepsin (>200 ng.mL^−1^). Abundant microaspiration was defined as the presence of pepsin at significant level in >74% of tracheal aspirates.

**Results:**

Amylase was measured in 1055 tracheal aspirates, collected from 109 patients. Using mean α-amylase level per patient, accuracy of α-amylase in diagnosing microaspiration was moderate (area under the receiver operator curve 0.72±0.05 [95%CI 0.61–0.83], for an α-amylase value of 1685 UI.L^−1^). However, when α-amylase levels, coming from all samples, were taken into account, area under the receiver operator curve was 0.56±0.05 [0.53–0.60]. Mean α-amylase level, and percentage of tracheal aspirates positive for α-amylase were significantly higher in patients with microaspiration, and in patients with abundant microaspiration compared with those with no microaspiration; and similar in patients with microaspiration compared with those with abundant microaspiration. α-amylase and pepsin were significantly correlated (r^2^ = 0.305, p = 0.001).

**Conclusion:**

Accuracy of mean α-amylase in diagnosing microaspiration is moderate. Further, when all α-amylase levels were taken into account, α-amylase was inaccurate in diagnosing microaspiration, compared with pepsin.

## Introduction

Despite the increased use of non-invasive ventilation, and high-flow nasal oxygen in patients with acute respiratory failure, intubation is still frequently performed in critically ill patients [1]. This procedure is associated with several complications, including ventilator-associated pneumonia (VAP), and tracheal ischemic lesions [2]. Although the incidence of VAP has probably decreased in the United States [3], this infection is still common in other countries [4], [5]. In addition, VAP is associated with prolonged duration of mechanical ventilation, and an overall attributable mortality of 13% [6]–[8].

Microaspiration of contaminated oropharyngeal and gastric secretions occur in a large proportion of intubated patients, and is a key factor in the pathogenesis of VAP [9]. Diagnosis of microaspiration in intubated critically-ill patients is important, in order to evaluate the efficiency of preventive measures aiming at decreasing the incidence of microaspiration, and VAP [10], [11]. Therefore, to test the efficiency of a new device aiming at preventing microaspiration, and VAP, it would be easier to first perform preliminary studies to evaluate the efficiency of such a device in preventing microaspiration before conduction large multicenter studies to test its impact on VAP incidence.

Several markers have been used to diagnose microaspiration of contaminated oropharyngeal, and gastric secretions in intubated critically ill patients, including technetium 99 m [12], blue dye [13], bile acids [14], and pepsin [9], [15]. However, several limitations of these markers preclude their routine use for clinical or experimental studies. Technetium 99 m is the gold standard for diagnosing microaspiration [12]. However, radioactive effects of this marker, and the need to transport the patient to the radiology department are major limitations for its routine use [10]. Blue dye is the most frequently used marker for aspiration [13], [16]. However, this marker is qualitative, and does not allow accurate quantification of the quantity of secretions aspirated in critically ill patients. Quantifying microaspirated secretions is important in this population, because the occurrence of lower respiratory infection is tightly correlated to the quantity of aspirated bacteria [17]. A pilot study, performed in 19 critically ill patients, found significantly higher concentration of bile acids in tracheal aspirates of patients with suspected VAP compared with those without VAP [14]. Further, the same group reported higher cocnentrations of bile acid in oropharyngeal secretions of patients with VAP compared with those without VAP [18]. However, further large studies are required to evaluate the accuracy of bile acids in diagnosing microaspiration and VAP. Pepsin was validated by several animal and human studies as an accurate marker of microaspiration of gastric content [9], [19]–[21]. However, this marker has a short window of detection of approximately 2 hours. In addition, pepsin does not allow diagnosing microaspiration of oropharyngeal secretions. Therefore, an accurate and easy to measure marker for microaspiration is still needed in critically ill patients.

Recently, α-amylase concentration in tracheal secretions, and bronchoalveolar lavage (BAL) was reported to be a potentially interesting marker to diagnose microaspiration and bacterial pneumonia [22], [23]. This marker is easy to measure in routine, and is not expensive. Moreover, α-amylase does not present the above-discussed limitations of other markers of microaspiration. However, to our knowledge, no study has evaluated the accuracy of amylase in diagnosing microaspiration compared to any other marker for microaspiration. Therefore, we conducted this retrospective study to determine the accuracy of amylase in diagnosing microaspiration, compared with pepsin.

## Patients and Methods

### Ethical Aspects

This retrospective study was performed in a single 10-bed medical ICU during an 11-month period. Patients included in this study were all included in a prior randomized study (ClinicalTrial.gov NCT01082666) [24] that was approved by the institutional review board (IRB) of the Lille university hospital. The current study was also approved by the same IRB. Written consent was obtained from the patients or their proxies for the current study.

### Inclusion and Exclusion Criteria

All patients included in our prior randomized controlled trial were eligible for this study, provided that at least one tracheal aspirate was available for α-amylase measurement. The only exclusion criterion was the impossibility of measurement of α-amylase, due to lack of sufficient quantity of tracheal secretions.

The initial randomized study [24] aimed to determine the impact of continuous control of cuff pressure on microaspiration of gastric contents. Patients >18 years, intubated and expected to require mechanical ventilation for at least 48 h were eligible for that study. Patients were excluded if they (a) were already enrolled in another trial, (b) had a contraindication for semirecumbent position, (c) had a contraindication for enteral nutrition, (d) had already undergone mechanical ventilation for >48 h at the time of screening for eligibility, (e) were admitted to the ICU with prior tracheostomy.

### Study Population

All study patients were intubated and mechanically ventilated for at least 48 hours. All tracheal tubes used in this study were high-volume low-pressure, and polyvinyl chloride-cuffed. Tracheal tube size was 7.5, and 8 in women and men; respectively. Tracheal cuff pressure was kept around 25 cmH_2_O using manual manometer or a pneumatic device.

Study patients received enteral nutrition according to a written protocol, including residual gastric volume measurement, and feeding interruption when gastric residual is >200 mL. Sucralfate was used for stress ulcer prophylaxis. Proton pump inhibitors were used to treat documented oesophagitis or gastric ulcer. Continuous subglottic suctioning was not utilized. Sedation was based on a written protocol including remifentanil and midazolam. Ramsay score was used to evaluate consciousness. The target Ramsay score was determined by the physicians. The bedside nurse adjusted sedative infusion to obtain target sedation level. A minimal positive end expiratory pressure of 5 cm H_2_O was applied to all patients. In all patients, tracheal suctioning was routinely performed by nurses using an open tracheal suction system. This procedure was performed 8 times daily or more if clinically indicated. Patients remained in semirecumbent position. Head-of-bed elevation was measured and adjusted by nurses 8 times daily (Target 45°). An Angle Indicator, designed to clearly display whether the head-of-bed was adequately elevated, was placed on side rails of all beds.

### Alpha Amylase and Pepsin Measurements

As part of the initial randomized controlled trial, pepsin was quantitatively measured in all tracheal aspirates during the 48 h following randomization. All tracheal aspirates were stored at −20°C which allowed subsequent measurement of α-amylase for the purpose of the current study [23].

Total amylase activity (salivary and pancreatic isoenzyme activity) and specific pancreatic amylase were measured in tracheal aspirates using commercially available kits (α-amylase EPS, and α-amylase EPS pancreatic respectively, from Roche Diagnostics, GmbH, Mannheim, Germany). Salivary amylase activity was calculated by the difference between total and pancreatic amylase activity.

Quantitative pepsin measurement was performed by an ELISA technique [24]. Briefly, polystyrene flat bottom microtiter plates were coated overnight at room temperature with 100 µl/well from each supernatant diluted two-fold in the coating buffer (PBS 0.1 M, pH 7.4). After wash steps, 100 µl of goat anti-pepsin antiserum (Interchim, Montluçon, France) diluted at 1∶2000 in PBS 0.1 M, pH 7.4 were added per well and incubated for two hours at 37°C. After washing, 100 µl/well of conjugate solution (alkaline phosphatase-labelled rabbit anti-goat IgG antiserum diluted at 1∶2000 in PBS 0.1 M, pH 7.4) were added and incubated for 1 hour at 37°C. The phosphatase alkaline activity was revealed by using *p-*nitrophenylphosphate as substrate. The concentration of pepsin in the tracheal aspirates was calculated from a standard calibration curve. Pepsin (EC 3.4.23.1) standards (25–400 ng/ml) were prepared by serial dilutions of a stock porcine gastric mucosa pepsin solution (100 µg/ml) (Merck, Darmstadt, Germany) in the coating buffer. The concentration of the stock solution used for standards was determined by means of the extinction coefficient of pepsin (E_mM = _51.3 at 278 nm). Pepsin was considered as positive at 200 ng/mL (25×8). Tracheal aspirates were very thick in several patients. Therefore all samples were first diluted using N-acetylcysteine (1/4), followed by dilution with coating buffer (1/2).

### Definitions and Data Collection

The primary objective of this study is to determine the accuracy of amylase in diagnosing microaspiration, compared with pepsin, in intubated critically ill patients.

Secondary objectives of this study were to determine the correlation between α-amylase and pepsin, and to compare α-amylase level and percentage of tracheal aspirates positive for α-amylase between patients with no microspiration, patients with microaspiration, and those with abundant microaspiration.

Microaspiration was defined as the presence of pepsin at significant level (>200 ng/mL) in at least one tracheal aspirate. Abundant microaspiration was defined as the presence of pepsin at significant level in more than 74% of tracheal aspirates (75^th^ quartile of percentage of tracheal aspirates positive for pepsin in study patients).

A tracheal aspirate was considered as positive for α-amylase if the level of α-amylase was >1688 UI.L^−1^ (Youden’s index, defined as the best sensitivity and specificity for α-amylase to diagnose microaspiration).

All data were prospectively collected. The following data were collected at ICU admission: age, male gender, simplified acute physiology score II, logistic organ dysfunction (LOD) score [25], comorbidities (diabetes mellitus, chronic heart failure, COPD, cirrhosis, chronic renal failure, immunosuppression, gastroesophaheal reflux), and causes for ICU admission. The following data were collected during ICU stay: duration of prior intubation, size of tracheal tube, and LOD score at randomization; cuff pressure, head-of bed elevation, quantity of enteral nutrition, vomiting, prokinetic drugs, proton pump inhibitor use, sedation, Ramsay score, Glasgow coma score, paralytic agent use, ventilatory mode, and positive end expiratory pressure during the 48 h following randomization.

### Statistical Methods

SPSS software (SPSS, Chicago, IL) was used for data analysis. Differences were considered significant if p<0.05. All P values were two-tailed. Categorical variables were described as frequencies (%). Normally distributed (Shapiro-wilk test) and skewed continuous variables were described as mean ± SD and median (interquartile range), respectively.

Patients were classified in three groups: no microaspiration, microaspiration, and abundant microaspiration. χ^2^ test, and Kruskal-Wallis test were used to compare qualitative and continuous variables between the three groups; respectively. If a significant difference was found between the three groups, further analyses were performed between each two groups. Appropriate corrections (Bonferoni) were made for multiple comparisons. For comparisons between each two groups, χ^2^ test or Fisher exact test were used to compare qualitative variables, as appropriate. Student t-test or Mann-Whitney U-test were used to compare normally distributed and skewed continuous variables, respectively.

To determine the accuracy of α-amylase in diagnosing microaspiration, the area under the receiver-operating characteristic curve ± SD was calculated. In addition, sensitivity, specificity, negative and positive predictive values were calculated. The cut-off value for α-amylase was the Youden’s index. These analyses were performed using the mean α-amylase values per patient. In addition, a second analysis was performed using α-amylase values coming from all samples, with adjustment for repeated measurements. This was done by using a generalized mixed model with the patient as random effect and the repeated measurements of α-amylase and microaspiration as independent and dependent variables, respectively.

Correlation between α-amylase and pepsin was analyzed by a Spearman test, and concordance was assumed by kappa coefficient.

## Results

**Figure 1:**
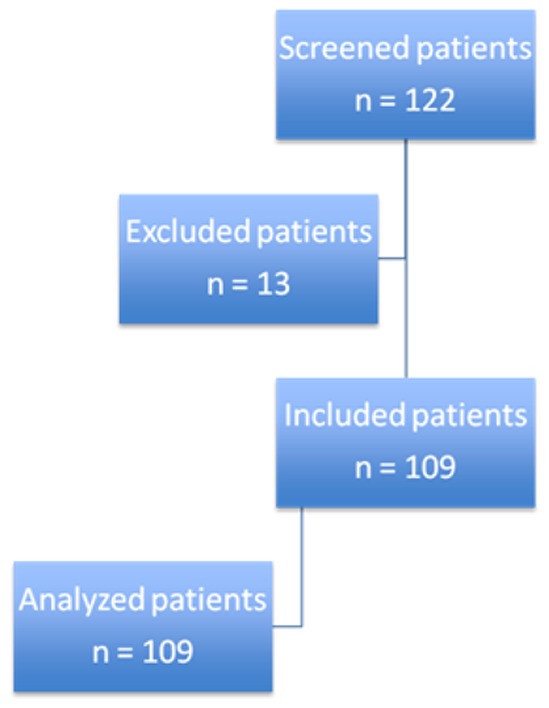
Study flowchart.

Amylase was measured in 1055 tracheal aspirates, representing 89.3% of the 1181 tracheal aspirates analyzed in the randomized trial. These tracheal aspirates were collected from 109 patients, representing 89% of the 122 patients included in the first trial. No sufficient quantity of tracheal secretions was available for α-amylase measurement in 126 specimens (11.2%) (Figure 1). No significant difference was found in patient characteristics between included patients, and those excluded because of insufficient quantity of secretions (data not shown). The median [IR] number of tracheal aspirates analyzed was 11 [8], [14] per patient. No significant difference was found in number of tracheal aspirates between patients with no microaspiration, patients with microaspiration, and those with abundant microaspiration.

Microaspiration was diagnosed in 55 (50%), and abundant microaspiration in 27 (24%) out of the 109 study patients. Patient characteristics are presented in Tables 1, and 2.

**Table 1:** Patient characteristics at ICU admission.

	No microaspiration n = 27	Microaspiration n = 55	Abundant microaspiration n = 27	P value
Age	59 (50–68)	58 (50–70)	66 (53–77)	0.244
Male gender	18 (66)	35 (63)	22 (81)	0.225
SAPS II	39 (30–54)	46 (34–52)	44 (35–46)	0.340
LOD score	4 (2–7)	5 (4–7)	5 (4–7)	0.135
Comorbidities				
Diabetes mellitus	4 (14)	8 (14)	3 (11)	0.899
Chronic heart failure	2 (7)	2 (3)	1 (3)	0.378
COPD	10 (37)	10 (18)	12 (44)#	0.030
Cirrhosis	3 (11)	3 (5)	3 (11)	0.562
Chronic renal failure	0 (0)	1 (1)	1 (3)	0.598
Immunosupression	8 (29)§	10 (18)	1(3)	0.042
Gastroesphageal reflux	2 (7)	3 (5)	0 (0)	0.390
Causes for ICU admission*				
Shock	13 (48)	19 (34)	7 (25)	0.226
ARDS	9 (33)	7 (12)	5 (18)	0.084
Community-acquired pneumonia	6 (22)	16 (29)	5 (18)	0.546
Hospital-acquired pneumonia	8 (29)	8 (14)	2 (7)	0.076
Healthcare-associated pneumonia	3 (11)	1 (1)	4 (14)	0.072
Neurologic failure	1 (3)	13 (23)	6 (22)	0.076
Acute exacerbation of COPD	3 (11)	4 (7)	5 (18)	0.311
Congestive heart failure	1 (3)	0 (0)	1(3)	0.354

Data are n (%) or median (interquartile range).

SAPS: simplified acute physiology score; LOD: logistic organ dysfunction; COPD: chronic obstructive pulmonary disease; ICU: intensive care unit, ARDS: acute respiratory distress syndrome.

# p<0.05 versus microaspiration,

§ p<0.05 versus abundant microaspiration.

*Several patients had more than one cause for ICU admission.

**Table 2:** Patient characteristics during ICU stay.

	No microaspiration n = 27	Microaspiration n = 55	Abundant microaspiration N = 27	p value
**At inclusion**				
Duration of prior intubation, d	2 (1–2)	1 (1–2)	1 (0–2)	0.364
Size of tracheal tube	8.0 (7.5–8)	8.0 (7.5–8)	8.0 (8–8)	0.318
LOD score	4 (1–6)	5 (3–8)	4 (2–6)	0.373
**During the 48** **h following inclusion**				
Pcuff, cmH_2_O	24 (23–27)	25 (22–26)	22 (22–25)	0.269
Head of bed elevation, angle achieved	43 (37–45)	42 (36–45)	37 (35–40)	0.237
Quantity of enteral nutrition, mL/d	750 (750–1000)	750 (750–1000)	750 (500–1000)	0.984
Vomiting	5 (18)	6 (10)	8 (29)	0.109
Prokinetic drugs	5 (18)	7 (12)	8 (29)	0.178
Proton pump inhibitor use	6 (22)	17 (30)	7 (25)	0.694
Sedation	18 (66)	32 (58)	15 (55)	0.674
Ramsay score	3 (2–4)	4 (2–4)	2 (2–4)	0.166
Glasgow score	10 (5–15)	7 (3–12)	9 (6–14)	0.280
Paralytic agent use	3 (11)	3 (5)	3 (11)	0.562
Ventilatory mode				0.962
ACV	20 (74)	42 (76)	20 (74)	
PSV	7 (25)	13 (23)	7 (25)	
Positive end expiratory pressure	7.5 (5–8)	6 (5–8)	5 (5–8)	0.445

Data are n (%) or median (interquartile range).

LOD: logistic organ dysfunction; Pcuff: cuff pressure; ACV: assist control ventilation, PSV: pressure support ventilation.

### Patient Characteristics

A significant difference was found between the three groups with regards to percentage of patients with COPD, and immunosuppression. The rate of COPD was significantly higher in patients with abundant microaspiration compared with those with microaspiration (p = 0.017, OR [95% CI] 3.6 [1.2–10]). The rate of immunosuppression was significantly lower in patients with abundant microaspiration compared with those with no microaspiration (p = 0.024, OR [95% CI] 0.09 [0.11–0.79]). No significant difference was found in COPD, or immunosuppression rate between other study groups. Other patient characteristics were similar in the three groups.

### Accuracy of α-amylase in Diagnosing Microaspiration and Abundant Microaspiration

**Figure 2:**
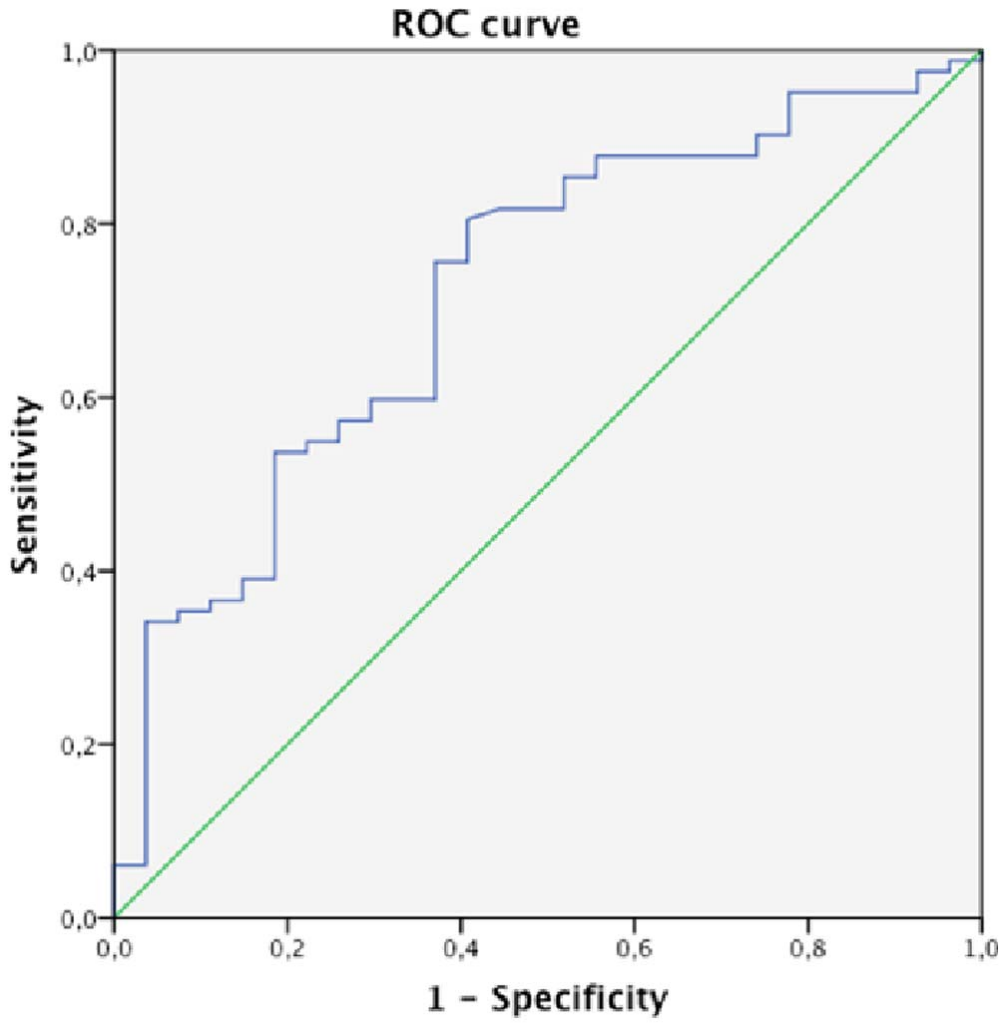
Accuracy of mean α-amylase in diagnosing microaspiration. Area under the receiver operator curve 0.72±0.05 [95% CI 0.61–0.83].

Using the mean α-amylase level per patient, the area under the receiver operator curve for the accuracy of α-amylase in diagnosing microaspiration was 0.72±0.05, [95% CI 0.61–0.83] (Figure 2). α-amylase value providing the best sensitivity and specificity to diagnose microaspiration was 1685 UI.L^−1^. This value provided the following characteristics for α-amylase in diagnosing microaspiration: sensitivity 0.87 (0.78–0.93), specificity 0.29 (0.13–0.50), positive predictive value 0.79, and negative predictive value 0.44.

**Figure 3:**
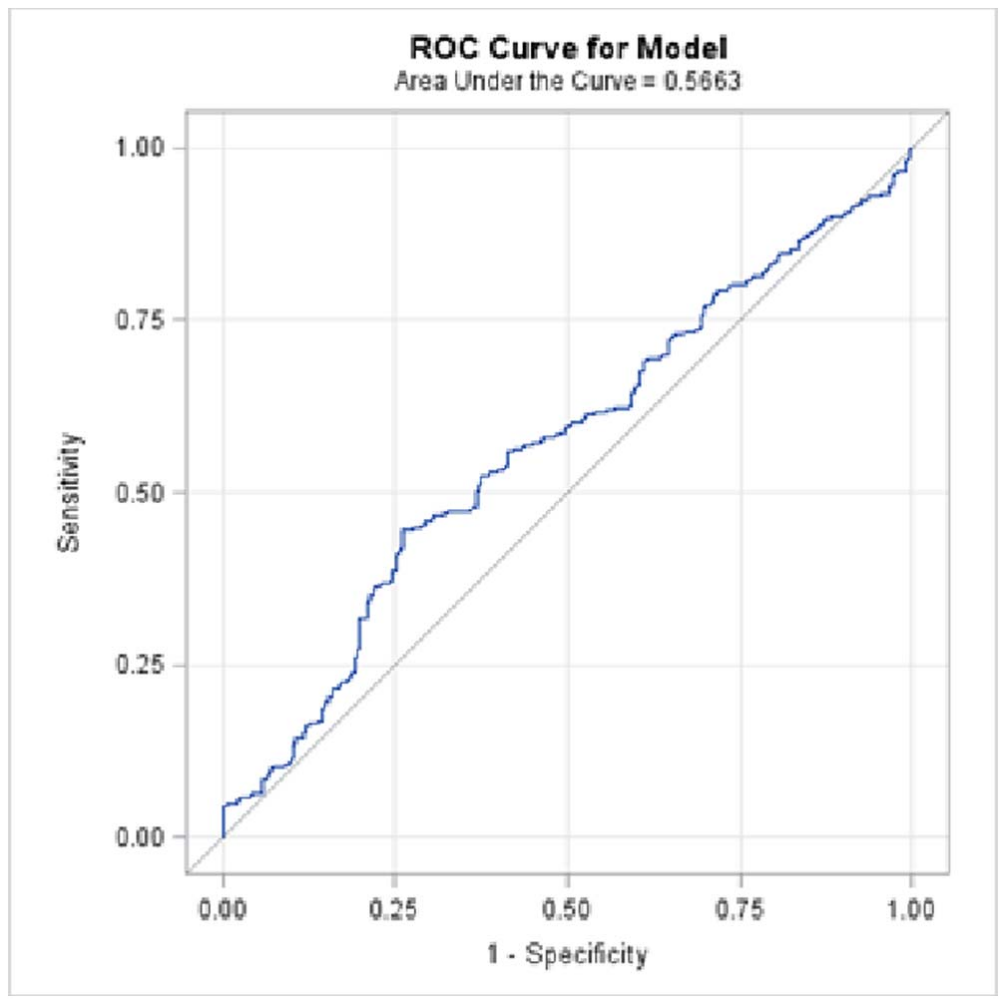
Accuracy of all α-amylase levels, coming from all tracheal aspirates, in diagnosing microaspiration. Area under the receiver operator curve 0.56±0.05 [95% CI 0.53–0.60].

Using all α-amylase levels, coming from all tracheal aspirates, and adjusting for repeated measurements, the area under the receiver operator curve for the accuracy of α-amylase in diagnosing microaspiration was 0.56±0.05, [95% CI 0.53–0.60] (Figure 3).

### Comparison of α-amylase Level between Different Study Groups

**Figure 4:**
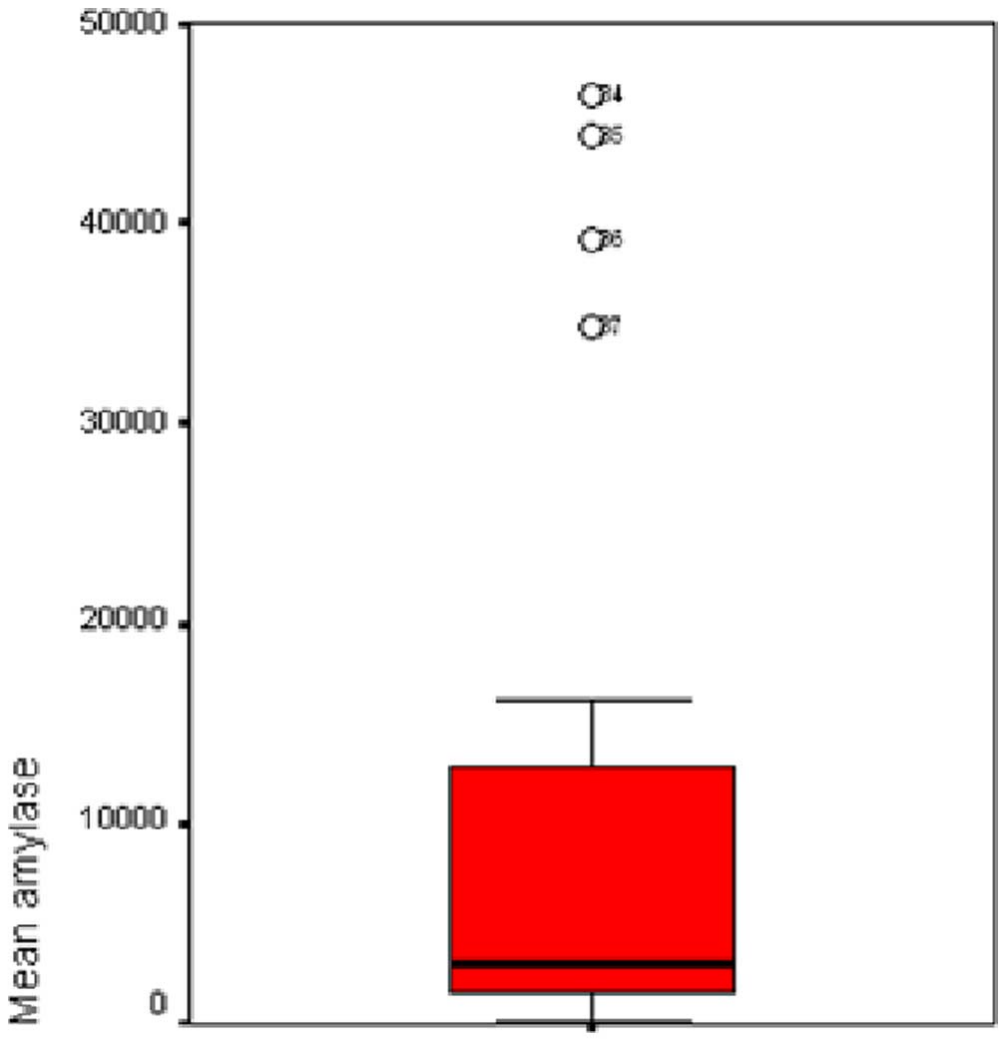
Mean α-amylase levels in patients with no microaspiration.

**Figure 5:**
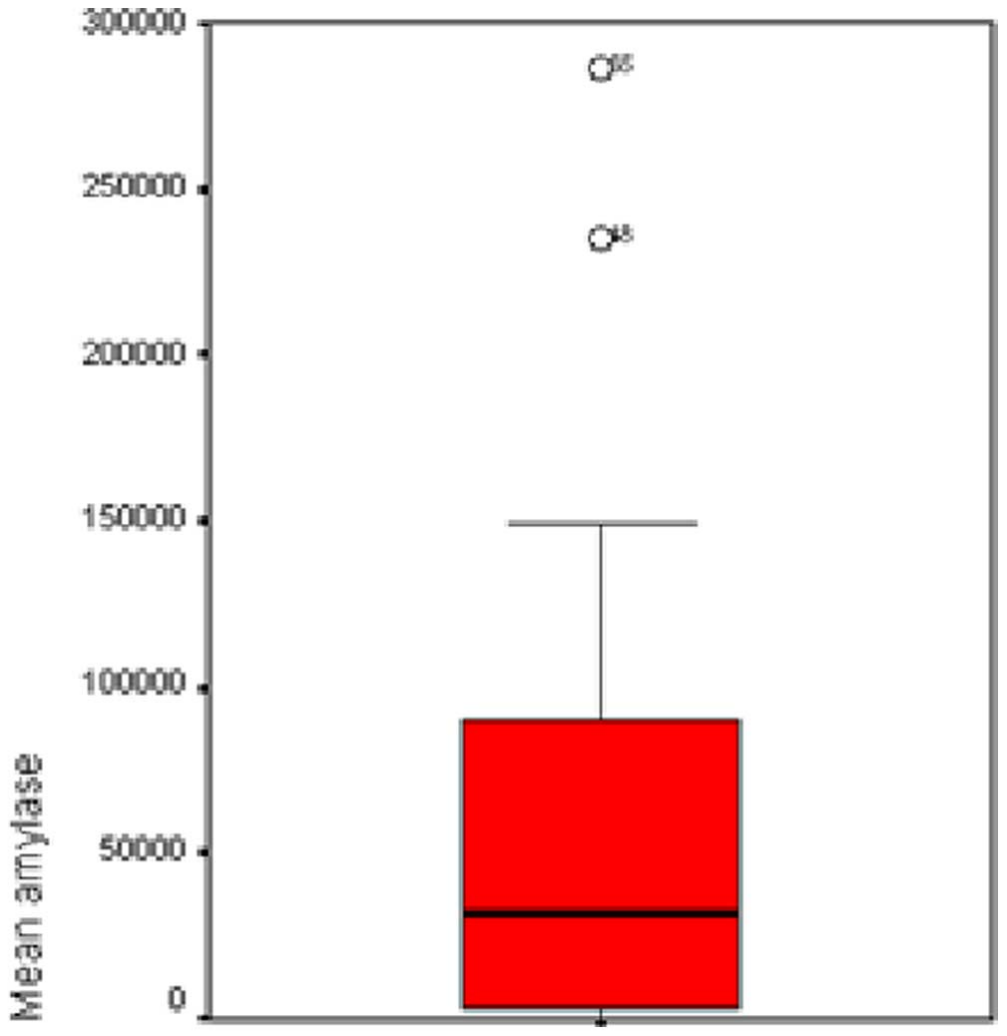
Mean α-amylase levels in patients with microaspiration.

**Figure 6:**
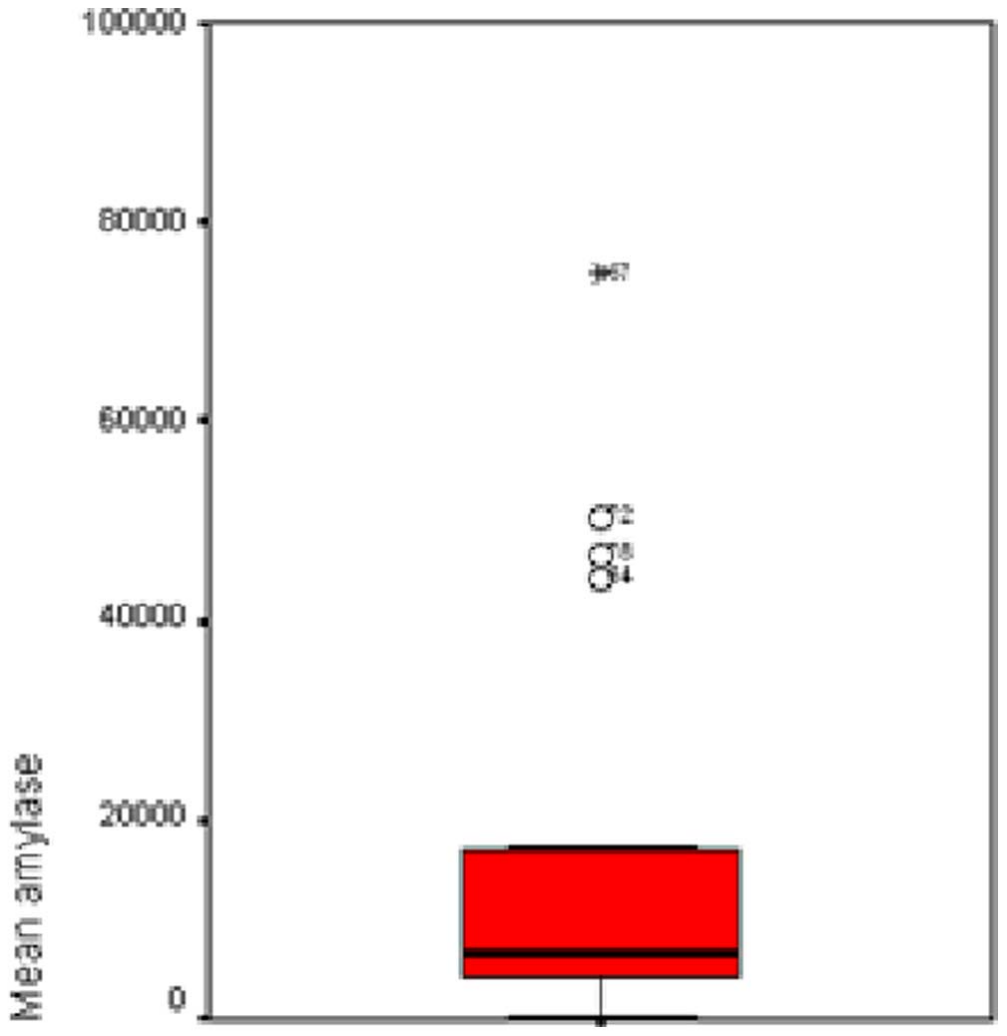
Mean α-amylase levels in patients with abundant microaspiration.

A significant difference was found in mean α-amylase level (Figures 4–6), and percentage of tracheal aspirates positive for α-amylase between the three groups. Mean α-amylase level, and percentage of tracheal aspirates positive for α-amylase were significantly higher in patients with microaspiration, and in patients with abundant microaspiration compared with those with no microaspiration. No significant difference was found in mean α-amylase level, or in percentage of tracheal aspirates positive for α-amylase between patients with microaspiration compared with those with abundant microaspiration (Table 3).

**Table 3:** Alpha amylase results.

	No microaspiration n = 27	Microaspiration n = 55	Abundant microaspiration n = 27	p value
Mean α-amylase				
Median (IQR)	3075 (1526–12796)# ^,^ §	22190 (4799–81443)	9771 (2100–60672)	0.001
Mini-Max	120–172466	146–635068	86–475660	
% of tracheal aspirateswith α-amylase >1685 UI/*				
Median (IQR)	62 (9–100)# ^,^ §	100 (83–100)	100 (31–100)	0.011
Mini-Max	0–100	0–100	0–100	

# p<0.05 versus microaspiration.

§ p<0.05 versus abundant microaspiration.

*Youden’s index.

### Correlation between α-amylase and Pepsin

**Figure 7:**
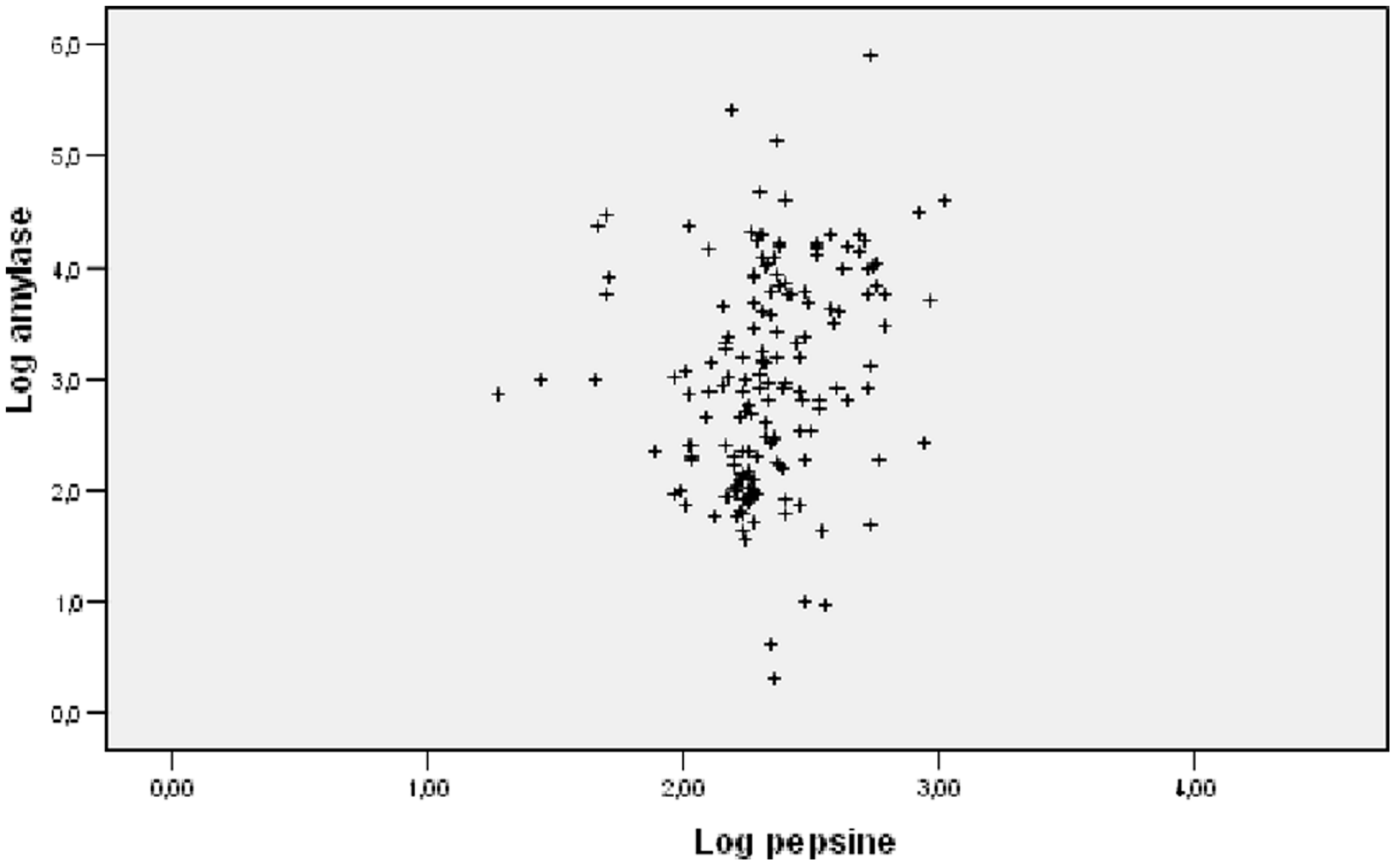
Correlation between α-amylase and pepsin. r^2^ =  O.305, P = 0.001.

α-amylase was significantly correlated to pepsin levels (r^2^ =  O.305, P = 0.001) (Figure 7).

## Discussion

The main findings of our study are the following: (1) accuracy of α-amylase in diagnosing microaspiration was moderate using the mean amylase level per patient, and low using all amylase levels. (2) Mean α-amylase level, and percentage of tracheal aspirates were significantly higher in patients with microaspiration, and in patients with abundant microaspiration compared with those with no microaspiration. (3) No significant difference was found in mean α-amylase level, and percentage of tracheal aspirates between patients with microaspiration and those with abundant microaspiration. (4) α-amylase was significantly correlated with pepsin, although this correlation was weak.

To the best of our knowledge, our study is the first to evaluate accuracy of α-amylase in diagnosing microaspiration in a large number of tracheal aspirates of critically-ill patients. Recently, two observational studies suggested that α-amylase measurement could be helpful in diagnosing aspiration pneumonia [22], and microaspiration [23] in critically-ill patients. Weiss and colleagues measured α-amylase in 296 BAL specimen from 280 patients with at least one risk factor for aspiration [22]. BAL amylase concentration increased with number of preintubation risk factors for aspiration. In addition, BAL amylase was significantly higher in patients with bacterial pneumonia. However, the ability of amylase in predicting bacterial pneumonia was moderate (area under the receiver operator curve 0.67 [95% CI 0.65–0.75]). Filloux and colleagues determined accuracy of α-amylase in diagnosing microaspiration in 26 patients intubated for >48 h (at high risk group), and 12 non intubated patients requiring BAL for different reasons (no microaspiration group) [23]. Tracheal amylase was significantly lower in the control group compared with the intubated group, and amylase gradually increased from tracheal, to subglottic, and oral samples. Interestingly, the cut-off value for α-amylase to diagnose microaspiration found in our study (1685 UI.L^−1^) was in line with that reported by these authors (1832 UI.L^−1^), in spite of different definition used for microaspiration.

The advantages in using α-amylase as a marker for microaspiration, compared with other markers are that α-amylase measurement is rapid, easy to perform, and cheap. However, the main drawback in using this marker is that microaspiration of gastric contents is not detected by α-amylase. The gastropulmonary route for entry of bacteria into the lower respiratory tract could be important in some patients [26]. The role of the stomach in microaspiration of contaminated secretions and subsequent VAP has been debated for a long period of time [27]. However, a prospective study using phenotyping of all bacterial strains isolated in oral, gastric, tracheal secretions, and BAL found both routes to be important in the pathogenesis of VAP [28].

Several factors could explain the poor performance of α-amylase in diagnosing microaspiration, and in differentiating abundant microaspiration from microaspiration. First, whilst α-amylase is a marker for microaspiration of oropharyngeal secretions, pepsin is a marker for microaspiration of gastric contents. In some patients, microaspiration of oropharyngeal secretions without microaspiration of gastric contents could have occurred. Second, the viscosity of oropharyngeal secretions and gastric contents is clearly different. Previous studies found viscosity to be an important factor influencing microaspiration of subglottic secretions through tracheal cuff [29]. Third, the detection window is different between these two markers, up to 72 hours for α-amylase [22] versus few hours for pepsin [9]. Therefore, the use of pepsin as a gold standard in our study has probably negatively affected the accuracy of α-amylase in diagnosing microaspiration.

Whilst COPD was associated with significantly higher rates of abundant microaspiration, immunosuppression was associated with significantly lower rates of microaspiration. COPD was identified as a risk factor for altered interaction between breathing and deglutition, resulting in deglutition abnormalities, and a higher risk for aspiration in this population [30], [31]. We compared factors that might have influenced microaspiration between patients with immunosuppression and those without immunosuppression (data not shown). The only significant difference between the two groups was younger age in immunosuppressed patients compared with those without immunosuppression. Advanced age was previously identified as a risk factor for aspiration and subsequent pneumonia [32]–[34]. However, the association between COPD, immunosuppression, and microaspiration was only found in univariate analysis, and a cause-to-effect relationship could not be confirmed.

Our study has some limitations. First, it was a retrospective single-center study. However, all data were prospectively collected. Second, markers for microaspiration were not measured during the whole period of mechanical ventilation. However, these measurements were performed during 48 h, representing 25% of median total duration of mechanical ventilation in study patients. Third, whilst the definition of microaspiration as the presence of pepsin at significant level in at least one tracheal aspirate is probably accurate, this definition only applies to microaspiration of gastric contents. Therefore, if another gold standard had been used, a better accuracy of α-amylase in diagnosing microaspiration could have been found. Further, the cut-off of 1688 UI.L^−1^ was based on the best sensitivity and specificity of α-amylase for diagnosing microaspiration in the same population. This might have artificially strengthened the results. However, we have repeated our analyses comparing % of tracheal aspirates positive for amylase between different study groups, using the cut-off reported by the above-discussed study [23]. The same results were obtained (data not shown), confirming that our cut-off of 1688 UI.L^−1^ is probably accurate. Fourth, we defined abundant microaspiration as the presence of pepsin in >74% of tracheal aspirates. Whilst this cut-off is the 75^th^ quartiles of % of tracheal aspirate positive for pepsin in study patients, this level was not validated by other studies. However, it is important to differentiate microaspiration from abundant microaspiration, because the occurrence of VAP is tightly correlated to the quantity of aspirated bacteria [17].

## Conclusion

Compared with pepsin, the accuracy of α-amylase in diagnosing microaspiration is low. Further prospective studies should compare α-amylase with other markers specific for microaspiration of oropharyngeal secretions, such as technetium 99, in order to determine the accuracy of amylase in diagnosing microaspiration.
